# Physicochemical Analysis of Composites Based on Yellow Clay, Hydroxyapatite, and *Clitoria ternatea* L. Obtained via Mechanochemical Method

**DOI:** 10.3390/ma18133011

**Published:** 2025-06-25

**Authors:** Klaudia Kowalska, Ewa Skwarek

**Affiliations:** Department of Radiochemistry and Environmental Chemistry, Institute of Chemical Sciences, Faculty of Chemistry, Maria Curie-Sklodowska University, 3 Maria Curie-Sklodowska Sq., 20-031 Lublin, Poland; klaudia.kowalska113@gmail.com

**Keywords:** yellow clay, hydroxyapatite, *Clitoria ternatea* L., composite materials, bioactive compounds

## Abstract

The present study describes the mechanochemical synthesis and physicochemical characterization of a novel composite material composed of yellow clay, hydroxyapatite, and *Clitoria ternatea* L. The synthesis was carried out using a solvent-free, energy-efficient mechanochemical method. The composite was analyzed for its toxicity, particle size distribution, release of bioactive compounds, surface morphology, structural features, and electrokinetic properties. UV-VIS spectrophotometry revealed that the release of bioactive substances was approximately 1.5 to 3 times higher in the composite compared to control samples. Particle size analysis indicated a wide distribution ranging from 350 to 1300 nm. Nitrogen adsorption–desorption (ASAP) confirmed the porous nature of the material, while SEM and FTIR analyses verified the successful incorporation of all components. Electrokinetic studies showed zeta potential values ranging from +15 mV to –32 mV, indicating varying colloidal stability. The proposed composite demonstrates promising potential as a carrier of biologically active substances for pharmaceutical and cosmetic applications.

## 1. Introduction

In recent years, there has been growing interest in the development of hybrid materials and composites based on natural and environmentally friendly raw materials. Such materials are gaining popularity due to their low toxicity, biocompatibility, biodegradability, and versatility in various applications, including environmental remediation, cosmetics, and biomedicine. One of the sustainable approaches for synthesizing such materials is mechanochemical synthesis, which does not require the use of solvents and allows for efficient incorporation of multiple components through mechanical energy input.

Clay materials are excellent adsorbents due to their large specific surface area in relation to particle size and their high cation exchange capacity. Clay is non-toxic, inexpensive, and widely available. Thanks to their surface reactivity in environmental matrices (soil and water), they can contribute to decontamination and recultivation processes through ion chemical interactions. Kaolinite, the primary component of yellow clay, is commonly found in soil and sedimentary rocks, and its addition can improve the mechanical stability of soils. Kaolinite is widely used in various industrial applications, including ceramics, pollutant adsorption, and oil drilling, due to its rheological properties [1].

Hydroxyapatite is a naturally occurring form of calcium apatite with the formula Ca_10_(PO_4_)_6_(OH)_2_. It makes up approximately 50% of human bone mass and is known for its excellent osseointegration and osteoconductive properties. Hydroxyapatite is mechanically stable under compressive forces, and its porous structure allows for applications in scaffolding and drug delivery systems. It can be used alone or as a component in composite materials, where it contributes to mechanical strength and biocompatibility [2].

*Clitoria ternatea* L. is a plant species known for its diverse bioactive compounds, including anthocyanins, flavonoids, and peptides, which demonstrate antioxidant, anti-inflammatory, and antibacterial properties [3]. It is easily cultivated and has a wide range of uses in food, cosmetics, and agriculture. Commercially available as a natural dye, it is also used in pest control in crops such as cotton and macadamia. Additionally, the plant has a long history of use in traditional Thai and Ayurvedic medicine, where its flowers are applied as natural remedies and cosmetic ingredients [4,5]. These properties make *Clitoria ternatea* L. an attractive biotechnological tool for the development of multifunctional materials.

Previous studies have explored clay–hydroxyapatite composites for applications in catalysis, environmental remediation, and biomedical uses. However, the combination of these inorganic matrices with plant-derived bioactive compounds, particularly *Clitoria ternatea* L., remains largely unexplored. Moreover, the use of mechanochemical synthesis in producing such hybrid composites offers new possibilities for environmentally friendly, scalable production with improved material properties. Mechanochemical processes are gaining increasing importance both in industry and environmental protection due to their numerous ecological and economic advantages. From an environmental perspective, mechanochemistry enables chemical reactions to be carried out without the use of solvents or with minimal solvent involvement, which significantly reduces the emission of toxic compounds and the amount of waste generated. For example, in catalyst production, mechanochemical methods can reduce solvent consumption by up to 90% compared to traditional solution-based syntheses. Additionally, mechanochemical reactions often proceed at room temperature or slightly elevated temperatures, lowering energy consumption by 30–50% compared to thermal processes. From an economic standpoint, mechanochemistry allows for shortening reaction times by up to half, which translates into higher production efficiency. The elimination or significant reduction of solvents decreases the costs of purchasing and disposing of chemical waste, which can account for as much as 20–40% of total operational costs in conventional processes. For instance, in pharmaceutical material production, mechanochemical methods have enabled cost reductions of approximately 15–25% while simultaneously reducing negative environmental impact. Mechanochemical technologies offer tangible environmental and economic benefits, making them an attractive alternative to conventional chemical processes, especially in the context of increasing demands for sustainable development.

The incorporation of biologically active plant components into clay–hydroxyapatite systems opens up new avenues for the controlled release of natural antioxidants such as chlorophyll A and B. These systems may serve as carriers in pharmaceutical or cosmetic formulations, with tailored physicochemical and electrochemical properties. Immobilizing bioactive agents in porous composite matrices enhances their stability and allows for more precise control over their release kinetics. As colloidal systems, their functional performance is closely related to electrokinetic stability, where surface charge density and zeta potential play key roles [6].

To date, no study has described a composite material combining yellow clay, hydroxyapatite, and *Clitoria ternatea* L. synthesized via a mechanochemical method. Therefore, the aim of this work is to synthesize and characterize such a composite and assess its physicochemical properties using a comprehensive set of analytical techniques. This study focuses on toxicity evaluation, chlorophyll concentration determination, particle size distribution, surface morphology (SEM), structural analysis (FTIR), specific surface area and porosity (ASAP), as well as electrokinetic parameters such as surface charge density and zeta potential [7].

## 2. Materials and Methods

### 2.1. Materials

Yellow clay (Mel-ok, Kyiv, Ukraine, hydroxyapatite (Thermo Scientific, Waltham, MA, USA), silica (Pilot plant of Chuiko Institute of Surface Chemistry, Kalush, Ukraine), TiO_2_ (Beurre 1–40%) (Beurre Cosmetic Ingredients, Kyiv, Ukraine), ZnO (Beurre 1–25%) (Beurre Cosmetic Ingredients), and *Clitoria ternatea* L. (Dary Natury, Grodzisk, Poland) were used to synthesize composites. The analyses were performed using ethanol (POCH p.a., Gliwice, Poland), NaCl (POCH p.a.), NaOH (POCH p.a.), and HNO_3_ (POCH p.a.).

### 2.2. Methods

The composite surface appearance was measured using SEM DualBeam microscope images (Thermo Fisher Scientific Inc., Waltham, MA, USA). The release of bioactive components was determined using a UV-VIS spectrophotometer: Cary 100, Cary 300 (Varian Instruments, Palo Alto, CA, USA). Particle size and zeta potential measurements were performed using a ZetaSizer 3000 (Malvern Instruments, Malvern, Great Britain). In the zeta potential calculations, the Smoluchowsky equation was also applied due to the κa of ~150 A of the NaCl solution with a concentration of 0.001 mol/dm^3^ that was prepared. Then, the prepared solution was added to a 250 cm^3^ beaker containing 0.02 g of the previously weighed solid sample. The obtained system was sonicated using the Misonix Sonicator XL2020 Ultrasonic Liquid Processor (SpectraLab Scientific Inc., Markham, ON, Canada) for 3 min, and then the pH values were set to 11, 10, 9, 8, 7, 6, 5 and 4. Surface charge density was determined using potentiometric titrations; pH_PZC_ was also determined using 0.001 mol/dm^3^ NaCl solution with two different adsorbent weights: 0.5 g and 1 g (due to the solubility of the samples). This was performed using a pHG201-8 glass electrode (indicator) (Radiometer, Warsaw, Poland) and an REF 451calomel electrode (reference) (Radiometer), applying the system for potentiometric tests. The titrant (NaOH solution) was dosed using an automatic burette, the Dosimat 665 by Metrohm (Opacz-Kolonia, Poland). The instruments were connected to a computer which collected data from the pH-meter and the burette and controlled the burette based on the pH changes recorded by the pH-meter. Each time, 50 cm^3^ of electrolyte and 0.2 cm^3^ of HCl were poured into the measuring cell in order to reduce the pH of the starting solution. We used the titr_v3 program (written for the needs of our research at the Department of Radiochemistry and Environmental Chemistry, UMCS). The ASAP 2420 device from Micromeritics Inc., Scientific (Eindhoven, Holand) was used to perform nitrogen adsorption and desorption. The specific surface area of the sample was calculated using the BET method, and porosity was determined using the Barrett, Joyner, and Halenda (BJH) procedure. The spectra were obtained using a Nicolet 8700A FTIR device coupled with a Nicolet NXR FT Raman spectrometer module from Thermo Scientific.

### 2.3. Mechanochemical Synthesis

Mechanochemical synthesis is an interesting alternative to solution chemistry. It involves grinding and all other types of mechanical action on samples. It eliminates the need to use solvents and thus reduces the production of environmentally hazardous organic waste. Mechanochemistry also opens new ways of obtaining products previously unavailable using solutions [8]. The knife mill used for synthesis operated at a power of 100–250 W. This type of mill does not consume significant amounts of electricity due to its short operation time and relatively low load. However, mechanical contamination, such as abrasion of the blades or container, is possible. As mentioned, a comprehensive life cycle assessment (LCA) would be the most suitable method to evaluate the overall sustainability of these processes. The synthesis of composites was carried out mechanochemically using a knife mill. The final effect of synthesis is a change in the organization of component particles and their accumulation. Hydroxyl groups present on the surface and adsorbed water can react with adsorbed particles. Mechanochemical synthesis produces a different structure than the starting materials. The consequence of this is intensive changes in the organization of its secondary particles forming the filler, which leads to the formation of nanoparticle aggregates and their agglomerates. There can be reactions involving surface hydroxyl groups of selected components and bound water during interaction with adsorbed plant particles. Composites (Table 1) were obtained using a knife mill with a capacity of 400 g and a speed of 24,000 RPM by mechanochemical synthesis. The previously weighed ingredients were mixed and ground in a knife grinder for 3 min.

## 3. Result and Discussion

### 3.1. Toxicity

When composites are potentially used in the medical, pharmaceutical, or cosmetic industries, it is necessary to become familiar with the potential toxicity of the main components of the obtained composites. The main percentage component of the composites is kaolinite contained in yellow clay. According to the data, cell viability in P388D1 cell cultures exposed to clay mineral samples changes only around 80 μg/cm^3^ [9].

The results of cell viability measured by the MTT test for hydroxyapatite nanoparticles indicate that for concentrations below 300 µg/cm^2^, there is no significant effect on the viability of cultured rat hepatocytes maintained for 72 h at a given concentration of hydroxyapatite [10]. In contrast, RAW264.7 cells were exposed to various concentrations of anthocyanin-rich parts of *Clitoria ternatea* L. flowers for 24 h. Significant changes were observed only for concentrations above 300 µg/cm^3^ [11].

### 3.2. Chlorophyll Concentration

**Figure 1:**
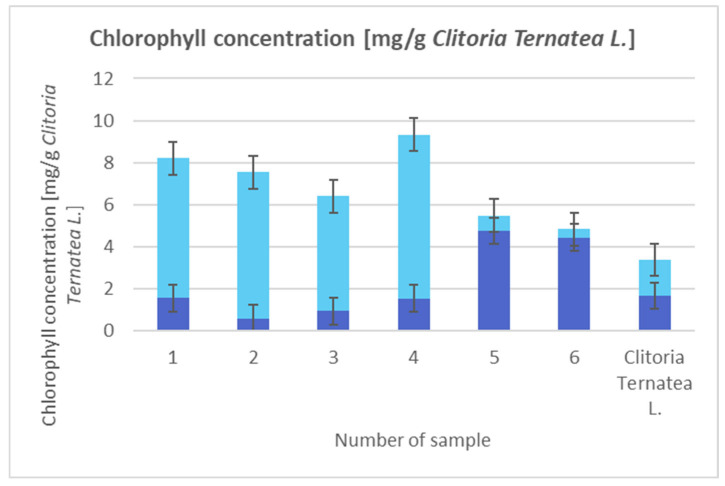
Concentration of chlorophyll A (navy blue) and B (blue).

To determine the chlorophyll concentration (Figure 1, 1 g of sample was measured into a 250 cm^3^ conical flask. Then, the samples were poured with 96% *v*/*v* ethanol, stirred, and left at room temperature. After one hour, the solution was centrifuged and measured by UV-VIS spectroscopy using a UV-VIS spectrometer (Varian Cary 100, Cary 300) at wavelengths of 649 and 665 nm. The concentration was calculated using the following formula (mg/g) [12]:

$$c_{A}=13.70\cdot A_{665}-5.76\cdot A_{649}$$

$$c_{B}=25.80\cdot A_{649}+20.04\cdot A_{665}$$

where

*A*—absorbance;

*C_A_*—chlorophyll *A* concentration;

*C_B_*—chlorophyll *B* concentration.

The chlorophyll content in the raw material, *Clitoria ternatea* L., is very diverse. For some raw materials, the discrepancy is even between 1 and 20 mg/g [13,14]. Regardless of the initial concentration, we can see a multi-critical increase in concentration, which is difficult to achieve for other types of improvements for biological materials. It was noticed that the chlorophyll concentration in the samples of the new composite was approximately 1.5 to 3 times higher than in pure *Clitoria ternatea* L.

The highest concentration of chlorophyll is found in sample 4 with the addition of hydroxyapatite (Figure 1). The concentration there is 7.8 mg/g. This can be explained by the fact that combining yellow clay with hydroxyapatite increases the specific surface area and pore size compared to pure clay. Additionally, weak hydrogen bonds are probably formed there between the components of the composite and chlorophylls. The consequence of this is better adsorption of chlorophylls and then their freer release. This is a result that is particularly important if we plan to use composites in cosmetic products.

### 3.3. Particle Size

**Figure 2:**
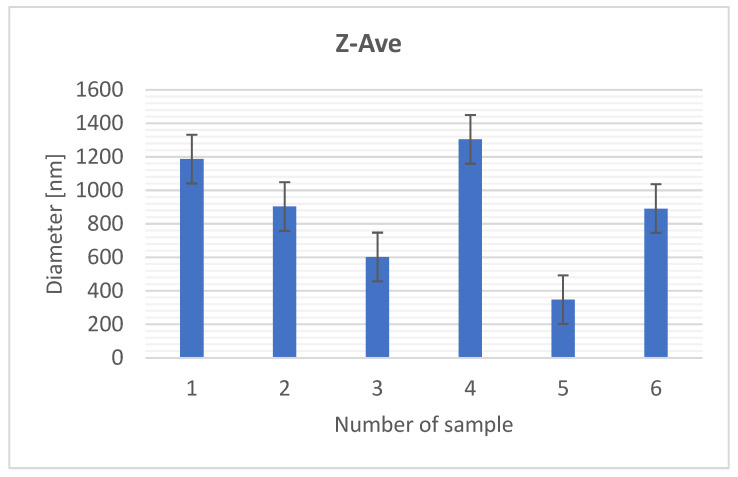
Average Z size.

To determine particle size (Figure 2), the averaged diameter of harmonic intensity particles (Z-Ave) was measured using a ZetaSizer 3000 (Malvern).

Figure 2 shows significant differences in the particle size of the composites, which range from 300 to 1350 nm. These differences allow the classification of the samples into nanocomposites and microcomposites, which suggests a different composition of the individual samples. Samples 1 and 4 are characterized by the largest measurement error, indicating a clearly different particle size. This may suggest the presence of both smaller and larger agglomerates, while sample 5 shows the smallest measurement error, which suggests a more homogeneous particle distribution.

The particle size measurement was performed wet, which may lead to aggregation of the particles due to intermolecular interactions, typical for such systems. Therefore, the aggregation process probably influenced the final particle distribution.

In the context of potential applications of composites, especially in cosmetics, it is worth paying attention to the ratio of the particle size to the skin’s ability to absorb them. Particles of up to 600 nm can penetrate into the deeper layers of the skin [15,16]. The average particle size of the tested composites is comparable to the particle size of other composites with organic additives. Many samples are within the optimum size range that allows for their absorption through the skin, and in the future, it is worth considering extending the time of mechanochemical synthesis, which may affect further adjustment of the particle size [12].

Mechanochemical synthesis plays a key role in shaping the morphology of the material. Depending on the proportions of the components, the grinding energy, and the process time, materials with a very diverse structure can be obtained. For cosmetic and pharmaceutical applications, smaller and more homogeneous particles, as in sample 5, may be particularly desirable due to better absorption and suspension stability. The high variability of particle size in samples 1 and 4 may indicate insufficient dispersion of the organic phase or incomplete homogenization of the components, which may affect the quality and efficiency of the final product.

### 3.4. Surface Analysis

**Figure 3:**
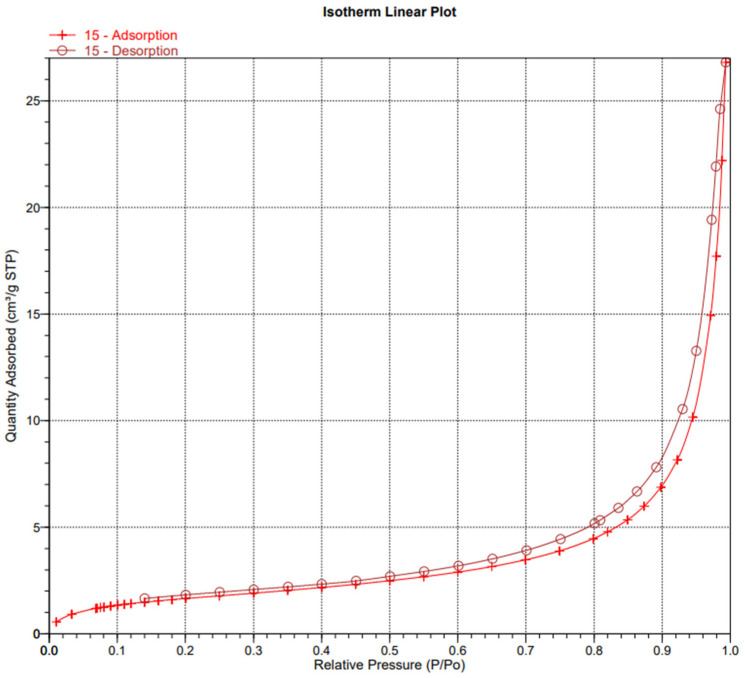
Isotherm linear plot of sample 1.

**Figure 4:**
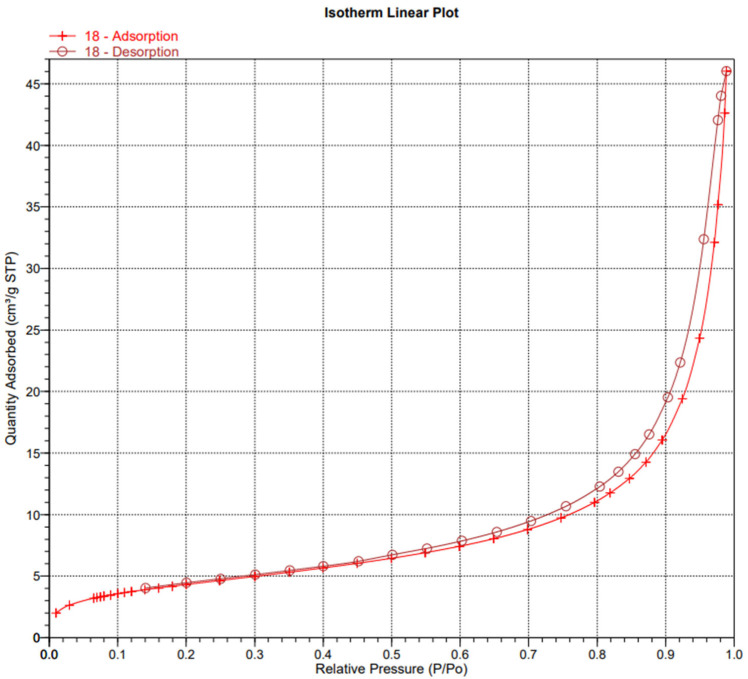
Isotherm linear plot of sample 4.

**Figure 5:**
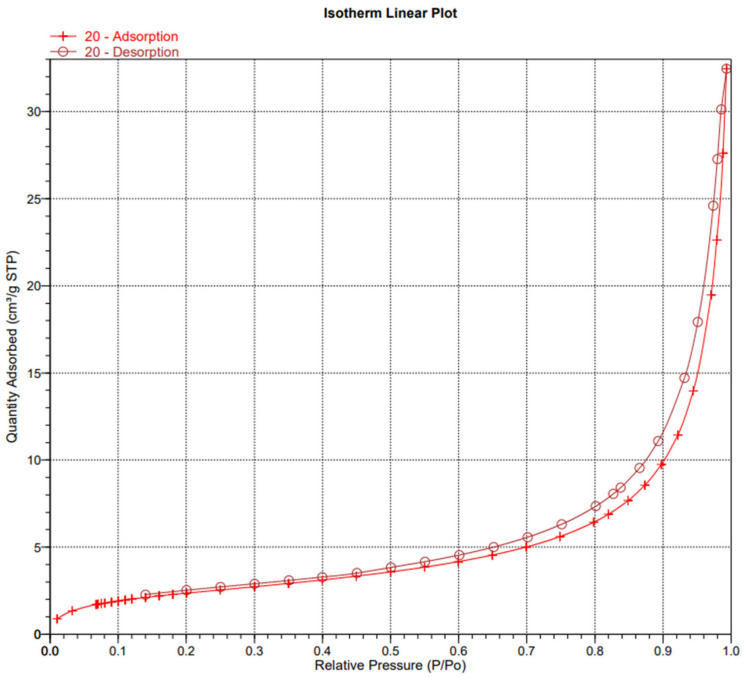
Isotherm linear plot of sample 6.

**Figure 6:**
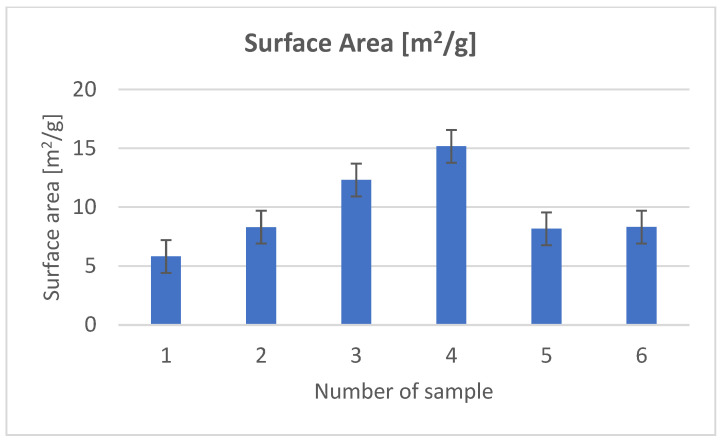
Surface area.

**Figure 7:**
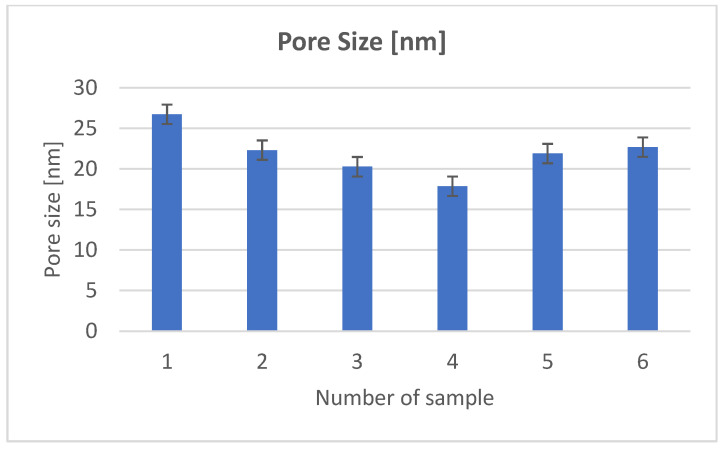
Pore size.

**Figure 8:**
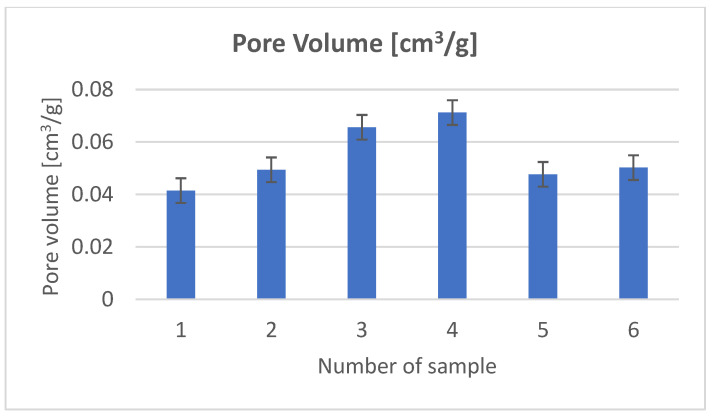
Pore volume.

In order to examine the structure of the composites, we used porosimetric tests (ASAP) (Figure 3, Figure 4, Figure 5, Figure 6, Figure 7 and Figure 8).

The surface area of the samples varied greatly, ranging from 6 to 15 m^2^/g, with the largest surface area found in the sample containing hydroxyapatite, silica, and *Clitoria ternatea* L. The pore size of the samples ranged from 18 to 27 nm, while the pore volume ranged from 0.04 to 0.07 cm^3^/g. The large pore volume provides excellent opportunities for the packing of biologically active substances. Pores of this size are classified as mesopores. The adsorption of biologically active substances, such as chlorophyll, in the mesopores of composite materials occurred in two stages: as pressure increased, the thickness of the thin adsorption layer gradually increased, typically being slightly thicker than on a flat surface (Figure 3, Figure 4 and Figure 5). The obtained adsorption isotherms in this case correspond to type IV according to the IUPAC classification (Figure 3, Figure 4 and Figure 5). This type of isotherm is characterized by a very gradual increase in the adsorbed amount in relation to the relative equilibrium pressure (p/p0) for values below 0.5. At higher pressures, the isotherm reaches a saturation plateau, which is a characteristic feature of mesoporous adsorbents where capillary condensation occurs. In this case, the desorption process is irreversible, as indicated by the desorption hysteresis related to adsorption. At a certain pressure, characteristic for the type and size of the pore, as well as the temperature and type of adsorbate, rapid condensation of the adsorbate occurs. During desorption, at a certain pressure value, the pores are quickly emptied of the adsorbate, leaving only a thin layer on the pore walls. It can be seen that the samples with the largest surface area and the largest pore volume also exhibit the smallest pore size. Comparing the data for the synthesized composites with two basic inorganic substrates (kaolinite and hydroxyapatite), we observe a smaller surface area and pore volume, along with an increase in pore size [17,18].

### 3.5. SEM-EXD

**Figure 9:**
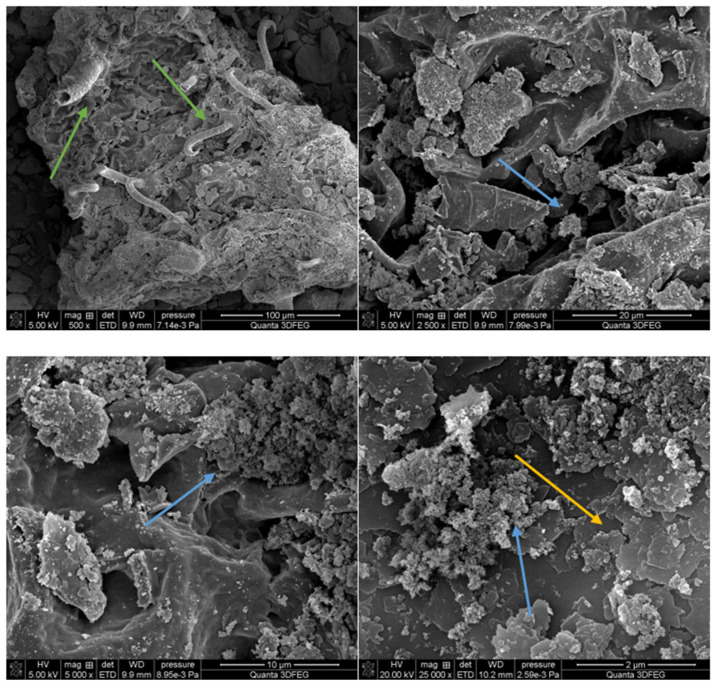
SEM photos of sample no. 4 at magnification from 500 to 25,000. Arrows: yellow—kaolinite; blue—hydroxyapatite; green—*Clitoria ternatea* L.

SEM-EXD images were taken (Figure 9). You can see lamellar structures of kaolinite, spongy hydroxyapatite scaffolds, and elongated, tubular structures that are particles of *Clitoria ternatea* L. flowers.

Based on the results of the grain size of the composites, which were in the range of 350 and 1300 nm, it can be confirmed from the SEM images that the composites are a micrometer in size. The grains of all the composites have irregular shapes and a porous structure can be seen on them, which is the result of the use of a shear mill for synthesis. The kinetic energy generated during the operation of the shear mill causes mass transfer, providing better contact between the surfaces of the composite materials. This leads to an increase in the surface energy and an increase in the chemical reactivity of the materials. High grinding energy has an impact on the occurrence of structural defects in the composites, destroying the crystalline structure of the clay and hydroxyapatite.

### 3.6. FTIR with KBr

**Figure 10:**
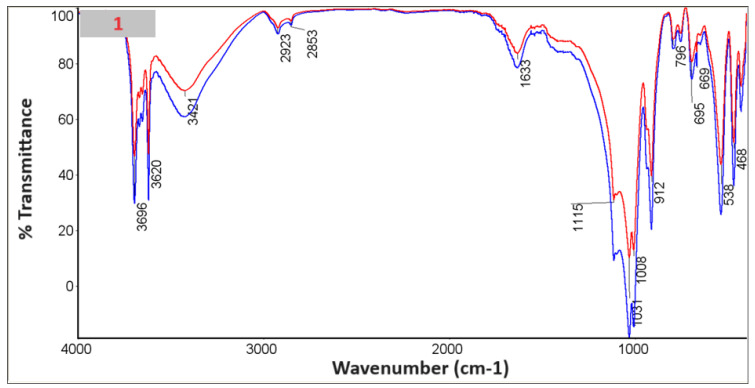
Spectrum of sample no. 1.

**Figure 11:**
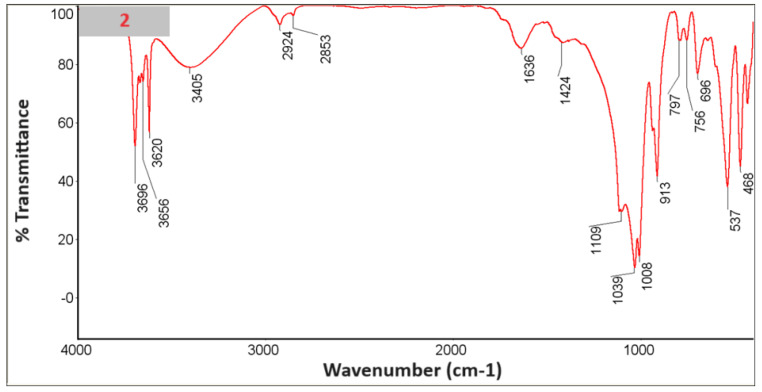
Spectrum of sample no. 2.

**Figure 12:**
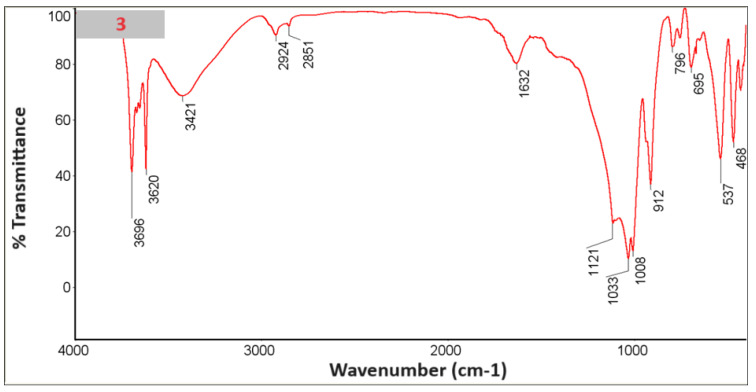
Spectrum of sample no. 3.

**Figure 13:**
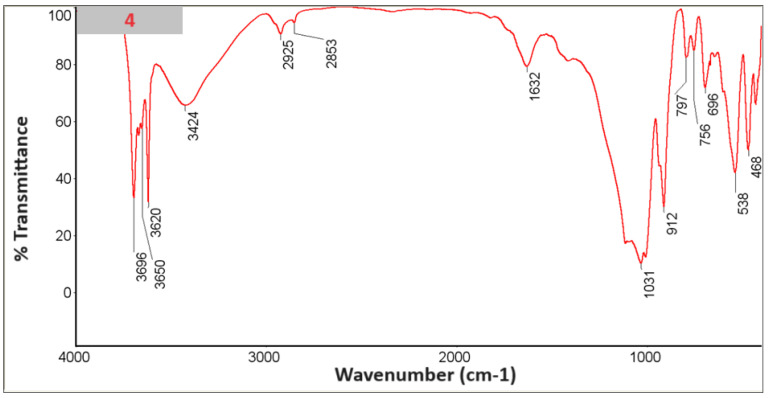
Spectrum of sample no. 4.

**Figure 14:**
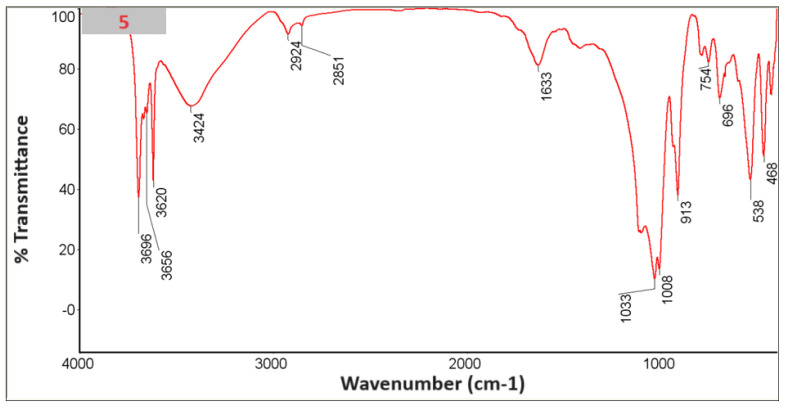
Spectrum of sample no. 5.

**Figure 15:**
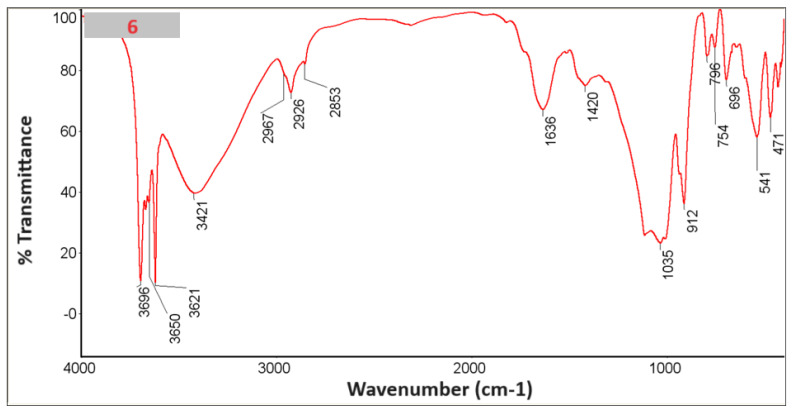
Spectrum of sample no. 6.

An FTIR study was performed with KBr (Figure 10, Figure 11, Figure 12, Figure 13, Figure 14 and Figure 15).

In the spectra, we can recognize bands that are characteristic of the composite components Table 2.

The presence of phosphate stretching at 472 nm and aromatic ring C–H distortion at 1047 nm coincides with the shift of the isoelectric point towards higher pH for the composites in whose spectra we recognized vibrations (samples 2, 4, 6). At the same time, for the given samples, the zeta potential for the pH range where the greatest stability occurs (pH between 6 and 12) shifts closer to zero, which may suggest lower stability [24,25].

### 3.7. Zeta Potential

**Figure 16:**
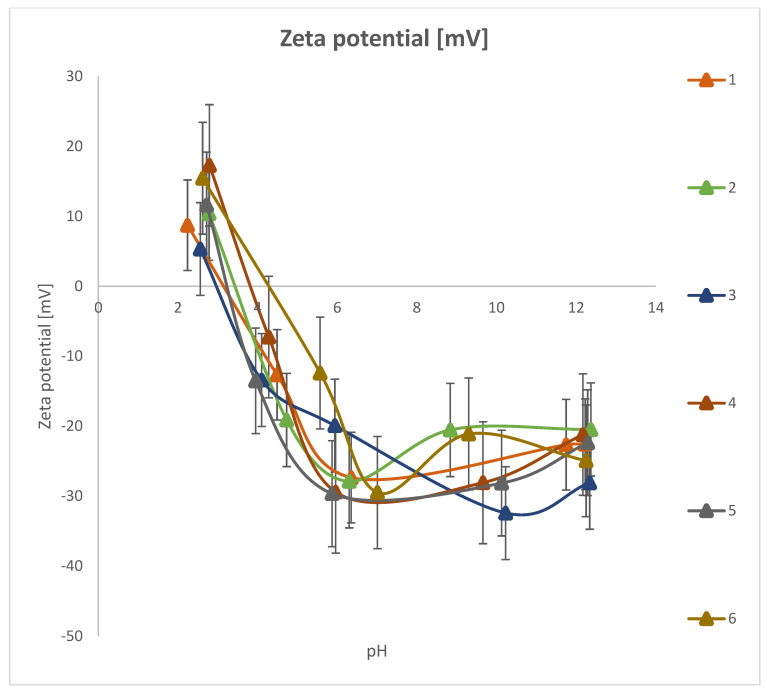
Zeta potential.

To measure the zeta potential (Figure 16), 0.01 g of the sample was weighed into a beaker and 250 cm^3^ of NaCl solution with a concentration of 0.001 mol/dm^3^ was added. The solution was exposed to ultrasound for 1 min. Then, using the ZetaSizer 3000 device (Malvern), the zeta potential of the samples was tested in the pH range from 2 to 12 using NaOH and HCl solutions with concentrations of 0.1 mol/dm^3^.

Figure 16 shows the zeta potential as a function of pH for six different composite samples. The data demonstrates clear pH-dependent behavior, with all samples exhibiting a sharp decrease in zeta potential from acidic to neutral pH, followed by a more stable trend at alkaline pH levels. These observations can be correlated with the specific composition of each sample (Table 1). Sample 1: This sample contains only yellow clay and *Clitoria ternatea* L. extract. It shows relatively high initial zeta potential values in acidic pH (~+15 mV), dropping significantly with increasing pH. The isoelectric point is located around pH 3–4. Its simple two-component composition suggests that the electrochemical behavior is mostly driven by the natural clay minerals and bioextract. Sample 2: The addition of 5% hydroxyapatite results in slightly reduced zeta potential in the acidic region, possibly due to the presence of calcium phosphate groups. Hydroxyapatite may also contribute to increased surface charge heterogeneity, slightly affecting stabilization in the alkaline range.

Sample 3: Incorporating 3% silica into the clay–bioextract matrix leads to a more negative zeta potential at higher pH. Silica is known for its strongly acidic silanol groups, which become deprotonated above pH 6, enhancing electrostatic repulsion. This is reflected in the lowest zeta potential values among the tested samples (reaching below –32 mV), indicating excellent colloidal stability. Sample 4: This composite, containing both hydroxyapatite and silica, displays a balance between the buffering effect of hydroxyapatite and the strong anionic character of silica. The zeta potential curve remains relatively stable and negative in the pH 6–12 range, which is favorable for dispersion stability. Sample 5: Here, 3% TiO_2_ replaces silica. TiO_2_, particularly in the anatase form, is amphoteric and exhibits complex surface charging behavior. The zeta potential profile of this sample fluctuates more in the alkaline range, suggesting possible surface reactivity or changes in particle interactions at higher pH. Sample 6: ZnO is known to exhibit positive surface charges in mildly acidic to neutral pH, which may explain the relatively higher zeta potential in the mid-pH range compared to other composites. However, in alkaline conditions, it behaves similarly to the other samples, showing stabilization in the –20 mV to –30 mV range. Across all samples, the sharp decline in zeta potential between pH 2 and 6 reflects the transition from protonated to deprotonated surface functional groups. The stabilization observed beyond pH 6 indicates that most particles acquire a strong negative surface charge, enhancing electrostatic repulsion and colloidal stability. The isoelectric points for all composites fall within pH 3 to 5, which is typical for systems containing clays, oxides, and plant-based additives. These findings highlight the role of surface-modifying agents—such as silica and metal oxides—in tuning the electrokinetic properties of clay-based composites, which is crucial for applications in drug delivery, wastewater treatment, and cosmetics.

The results of zeta potential measurements in the pH range from 2 to 6 showed that an increase in pH in this range causes a rapid decrease in the zeta potential value. In the pH range from 6 to 12, the results stabilize. Samples in the composite/electrolyte solution system are colloidally stable. The tested composites were characterized by zeta potential values from 15 mV to −32 mV. The isoelectric point (pHiep) is the isoelectric point, or pH value at which molecules or particles containing ionizing groups (e.g., proteins, nanoparticles, some composite materials) do not exhibit a net charge in the diffuse part of the electric double layer. At this point, the number of positive charges is equal to the number of negative charges, which means that the material has an electric charge of zero. It is a particularly important parameter in colloid and material chemistry, because it affects many physical and chemical properties of substances, such as colloid stability, interactions with surfaces and other substances, and pharmaceutical and cosmetic formulations. Samples 3 and 4 show the lowest zeta potential values in the pH range of 8–12 (below –30 mV), which indicates very good colloidal stability. These composites, containing silica (SiO_2_), can be particularly useful in applications requiring long-term dispersion, e.g., in drug delivery systems, filters, or nanocarriers. Recommended uses include pharmaceuticals, release control systems, and water purification.

The addition of hydroxyapatite to samples 2, 4, 5, and 6 reduces the zeta potential in the acidic range, which can improve biocompatibility and adsorption of calcium and phosphate ions. They can be used in the area of biomaterials, scaffolds for tissue engineering, and mineral cosmetics. Sample 6 contains zinc oxide, which effects a more positive zeta potential in the pH range of 5–8, which can increase the adhesion of cells or other negatively charged particles. This sample can be used in antimicrobial agents, protective cosmetics (UV filters), and dermatological preparations. Sample 5 with TiO_2_ shows zeta potential instability in alkaline environments, which may indicate surface variability depending on environmental conditions. This may be beneficial in applications requiring controlled surface reactivity. Its expected applications are as a photocatalyst, UV light-active material, and chemical sensor. *Clitoria ternatea* L. extract, present in all samples, may act as a stabilizing and antioxidant and also affect the bioactive properties of materials.

### 3.8. Point of Zero Charge (pHpzc)

**Figure 17:**
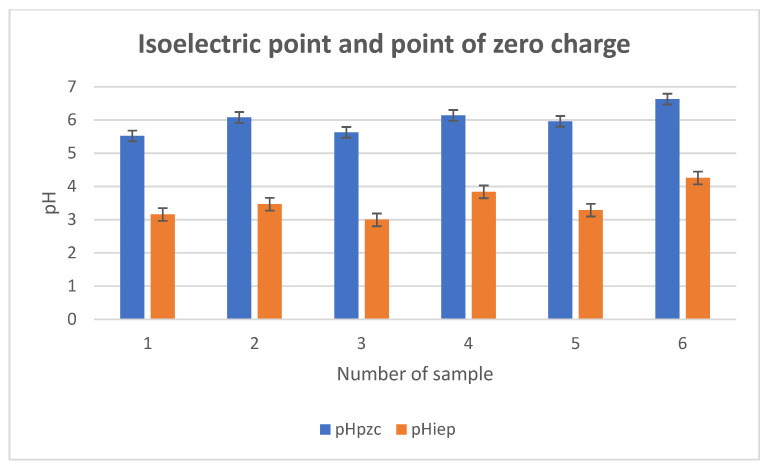
Isoelectric point (pHiep) and point of zero charge (pHpzc).

**Figure 18:**
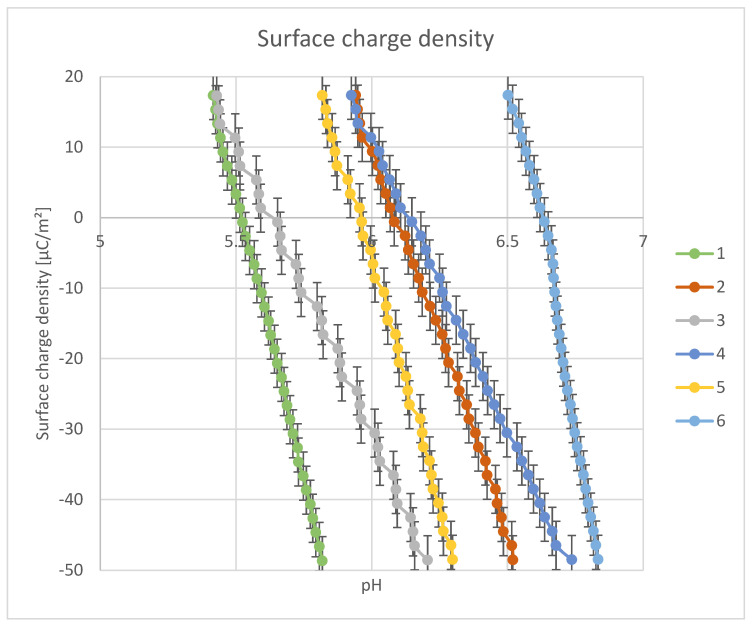
Surface charge density.

The point of zero charge (pHpzc) (Figure 17 and Figure 18) was determined using potentiometric titration.

Figure 17 shows the isoelectric point and point of zero charge values for the six tested composites. pH_IEP_ (orange bars) refers to the pH value at which the particles exhibit no net charge in the dispersed part of the electric double layer. This is the point at which the system is the least colloidally stable. pHpzc (blue bars) refers to the pH at which the total surface charge of the particles is zero (taking into account both the Stern and dispersed layers). For all samples, the pHpzc value is higher than the pHiep value. This indicates the occurrence of specific adsorption of anions (e.g., Cl^−^) on the surface of the particles, which lowers the pHiep value. This is typical for materials containing active surface groups (–OH, –COOH). Sample 6 (containing ZnO) is characterized by the highest values of both pHpzc and pHiep, confirming the basic nature of zinc oxide and its contribution to increasing the basicity of the material surface. Samples 3 and 5, containing silica (SiO_2_) and titanium oxide (TiO_2_), respectively, show the lowest pHiep values (~3.0–3.2), suggesting significant surface acidification, which may be beneficial for the adsorption of positive ions (e.g., heavy metals). Sample 2 (with hydroxyapatite) shows moderate pHpzc and pHiep values, which may be due to the surface heterogeneity of the material and the interaction of organic–mineral components. The large difference between pHpzc and pHiep indicates the material’s ability to specifically adsorb ions, which is particularly important in applications such as water purification (adsorption of metal ions), cosmetics (control of interactions with the epidermis), and biomaterials (interactions with proteins and cells). Materials with a low pHiep (e.g., samples 3 and 5) will be strongly negatively charged in neutral and alkaline conditions, which favors their colloidal stability and cation adsorption. Composite 6 can be used in slightly acidic environments, where the more alkaline nature of the surface (higher pHpzc) can improve biocompatibility and antibacterial activity (ZnO).

Figure 18 shows the dependence of the surface charge density (in μC/m^2^) on the solution pH for the six composites tested. The curves intersect the Y = 0 axis at different pH values, which correspond to the point of zero charge (pHpzc) for each sample. The surface charge density changes linearly with pH in the analyzed range (5–7), suggesting a well-expressed electrostatic relationship between pH and the ionization of surface groups. pHpzc is the intercept with the charge axis (Y = 0): for sample 1, it is approx. 5.4; sample 2: approx. 5.7; sample 3: approx. 5.3; sample 4: approx. 5.8; sample 5: approx. 6.0; sample 6: approx. 6.6. The increase in the content of metal oxides (TiO_2_, ZnO) causes a shift of the pHpzc towards higher values, which is consistent with the basic nature of these oxides, which increase the pKa of the surface groups. The lowest pHpzc is observed for samples containing only clay and/or silica (1 and 3), indicating a more acidic nature of the surface. Materials with a low pHpzc (below 5.5) will effectively adsorb positive ions in neutral and alkaline environments—useful for water purification from heavy metals (Pb^2+^, Cu^2+^). Materials with a high pHpzc (above 6.0), such as sample 6 (with ZnO), can be used where the adsorption of anions is desired (e.g., pharmaceuticals, dyes) or in lower-pH systems. The clear trend of pHpzc shift depending on the composite composition allows for targeted design of sorption materials, e.g., for a specific pH range of the target environment (water, cosmetics, soil, etc.).

## 4. Conclusions

Composites based on yellow hydroxyapatite clay and *Clitoria ternatea* L. were synthesized using the mechanochemical method. Based on the available literature, it is possible to ascertain the safety of the used substrates. An increase in chlorophyll concentration from 1.5 to 3 times, tested using UV-VIS spectrophotometry, proved the validity of using composites. The size of the particles classifies them between nanocomposites and microcomposites. This research also allowed us to deepen our knowledge about the rich surface of the newly synthesized products. Analysis of FTIR spectra and photos taken using an SEM microscope confirmed the presence of individual components of the composites and their correct incorporation into the whole. Electrochemical studies allowed us to determine the greatest stability in the range from 6 to 12 pH. Materials with a low pHpzc (<5.5) are effective for cation adsorption in neutral/alkaline environments. Composites containing TiO_2_ and ZnO exhibit a higher pHpzc, making them suitable for anionic contaminants. The tunable surface properties through composition adjustment allow for targeted design of sorbents for specific pH environments in applications like water treatment, cosmetics, and pharmaceuticals. The multitude of results shows that further research on this type of composite is not only necessary but also useful in the context of potential use, among others, in medicine, pharmacy, or the cosmetics industry.

## Figures and Tables

**Table 1:** Percentage composition by mass of composites.

Sample	Yellow Clay [%]	Hydroxyapatite [%]	Silica [%]	TiO_2_ [%]	ZnO [%]	*Clitoria ternatea* L. [%]
1	70	-	-	-	-	30
2	65	5	-	-	-	30
3	67	-	3	-	-	30
4	62	5	3	-	-	30
5	62	5	-	3	-	30
6	62	5	-	-	3	30

**Table 2:** List of bands characteristic of the spectra.

Wave Number [cm^−1^] ~	Type of Vibration	Component	1	2	3	4	5	6
**3696** **3653** **3620**	Al–O–H_str_	Kaolinite clay [17]						
**1115** **1031**	Si–O_str_							
**912**	Al–Al–OH_str_							
**796**	Al–Mg–OH_str_							
**755**	Si–O–Al_str_							
**696**	Si–O_str_, Si–O–Al_str_							
**541**	Si–O_str_, Si–O–Al_str_							
**471**	Si–O_str_							
**1628** **1421**	−C=C– in the structure of α, β unsaturated ketone, aromatic ring of phenolic and carbonyl group	*Clitoria ternatea* L. [19]						
**1047**	Aromatic ring C–H distortion							
**1648** **1455** **1417**	Carbonate_str_	Hydroxyapatite [20]						
**1091** **1042**	Phosphate_str_							
**472**	Phosphate_str_							
**910**	Si–O_str_	SiO_2_ [21]						
**800**	Si–O_str_							
**692**	Si–O–Si_str_							
**469**	O–Si–O_def_							
**798** **690**	Ti−O−Ti and Ti−O−C_bonds_	TiO_2_ [22]						
**2920-850**	Adsorbed on the ZnO nanoparticles	ZnO [23]						

Str—stretching, vib—vibration, def—deformational.

## Data Availability

The original contributions presented in this study are included in the article. Further inquiries can be directed to the corresponding author.
